# Changes of pro- and antioxidant indicators in experimental animals under acute small bowel obstructions

**DOI:** 10.25122/jml-2020-0066

**Published:** 2021

**Authors:** Svitlana Romanivna Pidruchna, Volodymyr Volodymyrovych Benedyct, Volodymyr Ivanovych Piatnochka, Nataliia Anatoliiivna Melnyk, Uliana Mykhailivna Zakharchuk

**Affiliations:** 1. Department of Medical Biochemistry, I. Horbachevsky Ternopil National Medical University, Ternopil, Ukraine; 3. Department of Surgery, Institute of Postgraduate Education, I. Horbachevsky Ternopil National Medical University, Ternopil, Ukraine; 2. Department of General Hygiene and Ecology, I. Horbachevsky Ternopil National Medical University, Ternopil, Ukraine; 4. Ternopil Regional Drug Treatment Clinic, Ternopil, Ukraine

**Keywords:** acute small bowel obstructions, lipid peroxidation, antioxidant protection system, APS – antioxidant protection system, ASBO – acute small bowel obstruction, DC – diene conjugates, LPO – lipid peroxidation, MA – malonic aldehyde, SOD – superoxide dismutase

## Abstract

Acute small bowel obstruction remains one of the most challenging nosologies in emergency surgery, leading to a pronounced imbalance between lipid peroxidation and the antioxidant defense system. We aimed to study changes in the anti- and prooxidant status of serum and small intestine wall in an experiment modeling acute small bowel obstruction. The control group included 11 rats, and the main group included 42 rats (simulation of mechanical bowel obstruction on day 1 was conducted in 14 rats, on day 2 – in 12 rats, on day 3 – in 16 rats). Acute small bowel obstruction was modeled by ligation. Serum analysis and removal of the small intestinal wall were performed on days 1, 2, and 3. Indicators of lipid peroxidation and antioxidant protection were determined by the spectrophotometric method, and the imbalance between lipid peroxidation and antioxidant protection gradually increased from 1 to 3 days of observation. On day 3, the low level of aldehyde increased 1.3 times, and the level of superoxide dismutase decreased 1.2 times compared to the control group. Pathophysiological changes in the wall of the small intestine are caused by the activation of lipid peroxidation and the exhaustion antioxidant protection, whereby the degree of their severity increases depending on the increase in time from the moment of modeling of acute obstruction of the small intestine.

## Introduction

Acute small bowel obstruction (ASBO) remains one of the most difficult nosologies in emergency surgery. Enteral insufficiency is a significant component of intestinal obstruction and endotoxicosis, which leads to multiple organ failure. One of the leading pathogenic links for the development of enteral insufficiency in ASBO is hypoxia, which develops as a result of impaired blood supply to the small intestine [1]. Hypoxia in the wall of the small intestine leads to destructive processes in the mucous membrane of the intestinal epithelium, which are initially manifested by vascular disorders, edema and plethora of the mucous membrane, a sharp expansion in it of capillaries and venules, along with aggregation of blood elements. Subsequently, the destruction of the mucous membrane and other layers of the intestinal wall follows [2].

One of the main causes of adverse effects in ASBO is peritonitis, which develops as a result of the progression of the pathological process in the absence of adequate intensive conservative and surgical treatment.

In recent years, researchers have paid increasing attention to the effects of oxidation, ischemia or reperfusion, and the development of oxidative stress on the functional state of the small intestine [3]. The study of pathophysiological patterns and tendencies of intracellular circulation disturbances is necessary for adequate and timely treatment.

Thus, studying the peculiarities of the processes of free radical oxidation and the state of the antioxidant protection system (APS) in ASBO is relevant, both from a theoretical and practical point of view.

We aimed to study the changes in the anti- and prooxidant status of serum and small intestine wall in an experiment modeling acute small bowel obstruction.

## Material and Methods

 The experiment was conducted on 53 white adult rats-males weighing 196–204 g, which were divided into two groups. The distribution data of experimental animals are shown in Table 1.

**Table 1.** Distribution of experimental animals.

**No. of experimental animals**	**Control group (1 group)**	**Mechanical bowel obstruction (2 groups)**		
		**1 day**	**2 days**	**3 days**
53	11	14	12	16

Group 1 – practically healthy intact animals; Group 2 – rats that were models of mechanical gut obstruction.

ASBO was modeled by ligation in the non-vascular area of the small intestine at a distance of 2 cm from the ileocecal junction.

All surgical interventions in experimental animals were performed under conditions of thiopental anesthesia by introducing into the abdominal cavity a solution of thiopental at the rate of 25 mg/kg body weight. At the end of the experiment, euthanasia of the animals was performed by rapid decapitation on days 1, 2, and 3.

The state of lipid peroxidation (LPO) at different times of the experiment was judged by levels of malonic aldehyde (MA) in the small intestine wall and diene conjugates in the blood plasma. The MA level was measured by the spectrophotometric method and expressed in mmol/L. The content of diene conjugates (DC) was also determined by the spectrophotometric method [4]. The content of DC was expressed in terms of extinction units (units).

The state of APS at different times of the experiment was judged by the levels of superoxide dismutase (SOD) activity, catalase activity of the small intestine wall, and SH-groups in blood plasma.

SOD activity of the tissue of the small intestine was determined on the basis of its ability to compete with nitrotetrazolium blue for superoxide anions using a spectrometric method [5].

The catalase activity of small intestine tissue was determined by a method whose principle is the ability of hydrogen peroxide to form a stable colored complex with ammonium molybdate using the spectrophotometric method. Also, the content of SH-groups was determined by the spectrophotometric method.

Statistical processing of digital data was performed by Excel and the STATISTICA software, using parametric and non-parametric methods of estimating the obtained data. The values of the arithmetic mean of the sample (M), its variance and the error of the mean (m) were calculated for all indicators. The significance of the difference of values between the independent quantitative values was determined at normal distribution by Student's t-test. In other cases, the Mann-Whitney U-test was used (differences at p<0.05 were considered significant).

## Results

A tendency for the intensification of LPO processes and a decrease in antioxidant protection resources, with a deepening of the established imbalance depending on the increase in the time of observation of the animals studied, was found while analyzing the data shown in Table 2. Significantly higher levels of MA and DC were found, as well as statistically significant lower rates of SOD activity, catalase, and the level of SH-groups in the studied animals with simulated mechanical intestinal obstruction compared with intact animals and the group of operated animals (p <0.05). Thus, the level of DC on the third day of the pathological condition in animals with experimental mechanical impassability was 32.07% higher compared to the first day of the experiment and by 13.02% compared to the second day. The MA level on the third observation day increased by 26.26% and 10.03% compared to the first and second days, respectively. There was a tendency to decrease the level of SOD with each passing day with experimental mechanical obstruction; therefore, on the third day, this indicator was significantly lower (by 20.53%) compared to the first day and by 8.93% compared to the second observation day. Regarding the activity of catalase and the content of SH-groups, there was a tendency to decrease the level of these indicators in accordance with the increase in the time of observation of the studied animals, but their value was unreliable.

**Table 2.** Indicators of LPO and ASP in animals with ASBO on days 1, 2, and 3 of the experiment.

Terms of observation (days)	**Diene conjugates (units)**	**Malonic aldehyde (μmol/g)**	**Catalase (H_2_O_2_ /min on 1 mg protein)**	**Superoxide dismutase (units/mg protein)**	**SH-groups (mmol/L)**
**I**	1.84±0.03	5.56±0.18	0.21±0.01	47.48±1.15	1.66±0.02
**II**	2.15±0.07 p I-II <0.001	6.38±0.21 p I-II <0.01	0.20±0.008 p I-II >0.05	41.43±1.82 p I-II <0.05	1.61±0.03 p I-II >0.05
**III**	2.43±0.07 p I-III <0.001 p II-III <0.01	7.02±0.16 p I-III <0.001 p II-III <0.05	0.19±0.007 p I-III >0.05 p II-III >0.05	37.73±0.44 p I-III <0.001 p II-III <0.05	1.55±0.07 p I-III >0.05 p II-III >0.05

p I-II – difference between observation days 1 and 2; p I-III – difference between observation days 1 and 3; p II-III –difference between observation days 2 and 3.

**Figure 1.**
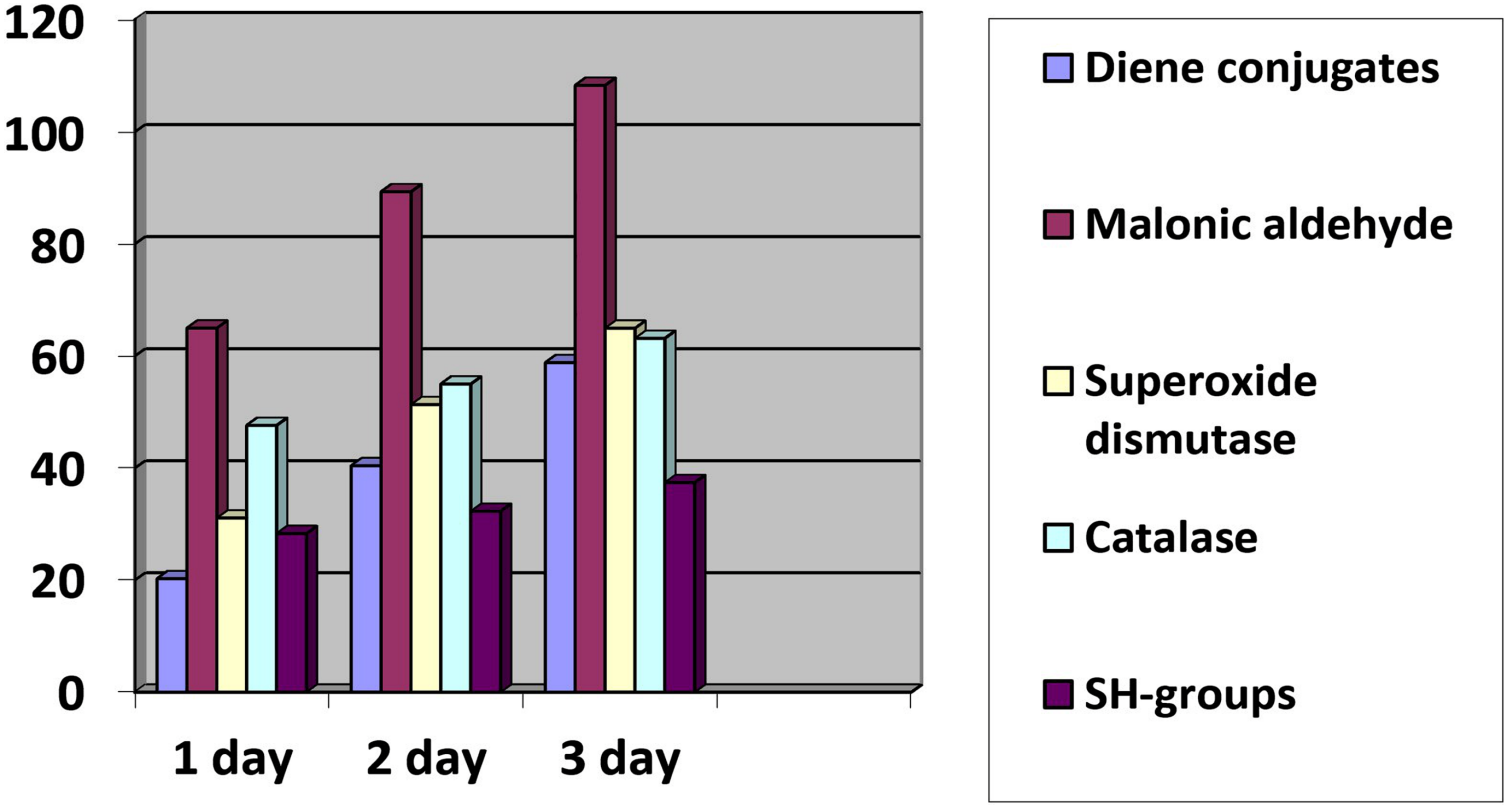
Percentage increase in LPO and APS in animals with ASBO.

Changes in LPO and APS are shown in Figure 1.

The analysis of the obtained data shows that the processes of oxidation are most significantly activated on the third day of observation. This group of animals also showed the most significant depletion of antioxidant protection resources.

In the analysis of the dynamics of the concentration of DC and MA in animals with experimental mechanical obstruction, an increase in the processes of LPO was observed in accordance with the increase in the observation time – the longer the period from the moment of the ASBO modeling, the higher the concentration of lipoperoxidation indicators. This can be explained by the increase in cell wall destruction with the simultaneous activation of all LPO units after an injury to the small intestinal wall. Simultaneously, there is a simultaneous formation of both initial products of lipoperoxidation (DC content) and its final products (MA content).

Regarding antioxidant protection, there was a rapid increase in the level of SOD activity, depending on the increase in the observation period with simulated mechanical intestinal obstruction. Activation of the antioxidant system can be explained by an increase in the adaptive-regulatory processes of the organism of the animals studied.

Analyzing the data obtained, we found that laparotomy – a stressful situation that does not significantly alter the antioxidant system – increases the LPO processes. This can be explained by the structural and functional disorders of the small intestine wall and the imbalance of local homeostasis regulation that occurs at the site of surgery. However, it should be noted that the overall condition of the experimental animals did not change.

## Disscusion

With the development of ASBO, there is an increase in intra-abdominal pressure, which leads to the development of ischemic-reperfusion syndrome; there is an increase in reactive oxygen species and damage to cell membranes, which ultimately leads to necrosis. Activation of lipid peroxidation processes leads to an imbalance in the APS, which exacerbates the development of endogenous intoxication and enteral insufficiency syndromes [6–7]. Increasing the production of free radicals reduces the protective properties of the intestinal mucosa and facilitates bacterial translocation, naturally increasing the risk of failure of intestinal anastomoses. Moreover, the latter is an important cause of postoperative mortality [8].

Most often in the literature, there is an assessment of the intensity of free-radical processes on the concentration of products of intermediate and terminal peroxidase catalysis: MA, DC, ketodienes and trienes. The antioxidant system is studied for the activity of the SOD and catalase enzymes and indicators of the non-enzymatic system of APS – the content of reduced glutathione (SH-groups) [9–10].

Based on our research results, we have obtained data that are consistent with those of other studies. In particular, there is a trend towards intensified lipoperoxidation processes and decreased antioxidant protection resources. Statistically higher levels of MA and DC were found, as well as statistically significant lower rates of SOD activity, catalase, and the level of SH-groups in the studied animals with simulated mechanical intestinal obstruction compared with intact animals and the group of operated animals (p <0.05). Thus, the level of DC on the third day of the pathological condition in animals with experimental mechanical obstruction was 32.07% higher than on the first day of the experiment and by 13.02% than on the second day. The MA level on the third observation day increased by 26.26% and 10.03% compared to the first and second days, respectively. There was a tendency towards a decreased level of SOD with each passing day of experimental mechanical obstruction. So, on the third day, this indicator was statistically significantly lower – by 20.53% – compared to the first day and by 8.93% compared to the second observation day. Regarding the activity of catalase and the content of SH-groups, there was a tendency towards a decreased level of these indicators in accordance with the increase in the time of observation of the studied animals, but their value was unreliable.

According to some studies, surgery increases the intensity of LPO, which promotes the entry into the bloodstream of unoxidized breakdown products [11]. However, the status and activity of APS blood do not always objectively reflect changes in organs and systems.

According to some authors, the activity of SOD and catalase increases by one day from the beginning of obstruction with a subsequent tendency towards a decrease by the second and third days, which indicates the failure of APS cells and the irreversibility of ischemic cell damage by LPO products [12]. In our research, the activity of SOD and catalase decreased slightly after laparotomy, which obviously indicates a greater compensatory capacity of the APS of the animals studied in the experiment. In this regard, it is necessary to study more deeply the changes in the APS system and free-radical processes that occur not only in the blood because of ASBO but also in organs and tissues [13].

Attention must be drawn to the fact that most scientific studies note the importance of changes occurring in the APS systems of the body during ASBO [14], their impact on disease prognosis and postoperative mortality, and also the need for preventive and corrective therapy.

## Conslusion

Pathophysiological changes in the small intestine wall are caused by the activation of lipid peroxidation and the exhaustion antioxidant protection, whereby the degree of their severity increases depending on the increase in time from the moment of modeling the acute obstruction of the small intestine. This indicates a statistically significant increase in malonic aldehyde content on day 3 of the experiment by 26.26% compared to day 1, as well as a decrease in superoxide dismutase activity by 20.53%.

## Acknowledgments

This work is a fragment of the planned research work – Development of new open (mini-access) and laparoscopic surgical interventions in the treatment of diseases of the abdominal cavity on the principles of the multimodal program “fast track surgery” (State registration number: 0119U002805).

### Conflict of interest

The authors declare that there is no conflict of interest.
